# Mechanical Cues, E-Cadherin Expression and Cell “Sociality” Are Crucial Crossroads in Determining Pancreatic Ductal Adenocarcinoma Cells Behavior

**DOI:** 10.3390/cells11081318

**Published:** 2022-04-13

**Authors:** Francesca Bianchi, Michele Sommariva, Laura Brigida Cornaghi, Luca Denti, Ambra Nava, Francesca Arnaboldi, Claudia Moscheni, Nicoletta Gagliano

**Affiliations:** 1 Department of Biomedical Sciences for Health, Università degli Studi di Milano, 20133 Milan, Italy; francesca.bianchi1@unimi.it (F.B.); michele.sommariva@unimi.it (M.S.); laura.cornaghi@unimi.it (L.B.C.); ambra.nava01@universitadipavia.it (A.N.); francesca.arnaboldi1@unimi.it (F.A.); 2 U. O. Laboratorio Morfologia Umana Applicata, IRCCS Policlinico San Donato, San Donato Milanese, 20097 Milan, Italy; 3 Department of Biomedical, Surgical and Dental Sciences, Università degli Studi di Milano, 20133 Milan, Italy; luca.denti1@unimi.it; 4 Department of Biomedical and Clinical Sciences “L. Sacco”, Università degli Studi di Milano, 20157 Milan, Italy; claudia.moscheni@unimi.it

**Keywords:** pancreatic cancer, 3D spheroids, actin cytoskeleton, E-cadherin, epithelial-to-mesenchymal transition, MMPs, mechanotransduction, YAP

## Abstract

E-cadherin, an epithelial-to-mesenchymal transition (EMT) marker, is coupled to actin cytoskeleton and distributes cell forces acting on cells. Since YAP transduces mechanical signals involving actin cytoskeleton, we aimed to investigate the relationship between YAP and mechanical cues in pancreatic ductal adenocarcinoma (PDAC) cell lines, characterized by different EMT-related phenotypes, cultured in 2D monolayers and 3D spheroids. We observed that the YAP/p-YAP ratio was reduced in HPAC and MIA PaCa-2 cell lines and remained unchanged in BxPC-3 cells when cultured in a 3D setting. CTGF and CYR61 gene expression were down-regulated in all PDAC 3D compared to 2D cultures, without any significant effect following actin cytoskeleton inhibition by Cytochalasin B (CyB) treatment. Moreover, LATS1 mRNA, indicating the activation of the Hippo pathway, was not influenced by CyB and differed in all PDAC cell lines having different EMT-related phenotype but a similar pattern of CTGF and CYR61 expression. Although the role of YAP modulation in response to mechanical cues in cancer cells remains to be completely elucidated, our results suggest that cell arrangement and phenotype can determine variable outcomes to mechanical stimuli in PDAC cells. Moreover, it is possible to speculate that YAP and Hippo pathways may act as parallel and not exclusive inputs that, converging at some points, may impact cell behavior.

## 1. Introduction

Increasing evidence has demonstrated that three-dimensional (3D) in vitro culture systems, such as 3D spheroids, recapitulate the in vivo tissue architecture and cell–cell contacts and differentiation patterns [1,2,3,4], offering the possibility to investigate important aspects of tumor biology and pathophysiology and to discover new therapeutic targets [5,6,7]. Cells grown in 3D spheroids not only represent a more reliable experimental setting compared to 2D monolayers, but they also retain a “social behavior” based on their cell–cell interactions mediated by intercellular junctions.

In epithelial cells, cell–cell junctions are specialization located on the lateral domain of the plasma membrane, engaging transmembrane proteins that have their extracellular domain connecting apposed cells and maintaining tissue integrity. E-cadherin is a 120 kDa molecular weight transmembrane protein of adherens junctions that anchors epithelial cells [8]. It is considered the prototypical and major determinant of the epithelial phenotype since functional adherens junctions in epithelial cells are needed to preserve cell polarity and tissue integrity, and contact inhibition as well [9,10]. The E-cadherin cytoplasmic domain interacts with β-, α- and P120-catenin molecules that stabilize E-cadherin at the plasma membrane and mediate interactions with the actin cytoskeleton. α-catenin links the cadherin molecular complex to F-actin by directly binding actin filaments by vinculin (VNC) [11,12].

E-cadherin down regulation is a key marker of epithelial-to-mesenchymal transition (EMT), a step-wise process described both in normal and tumor cells providing cancer cells with a metastatic phenotype [13,14]. During EMT, the loss of “epithelial” phenotype and the acquisition of a “mesenchymal” phenotype by epithelial cells is related to the down-regulation of epithelial markers and the up-regulation of mesenchymal ones [15,16]. Epithelial cells undergoing EMT can experience the so-called “cadherin switch”, characterized by E-cadherin downregulation paralleled by an increased N-cadherin expression [17,18,19], a process frequently observed in cancer, such as in poorly differentiated pancreatic ductal adenocarcinoma (PDAC) cells [18,19,20,21].

Mechanotransduction is the ability of cells to translate mechanical stimuli into biochemical signals that can ultimately influence gene expression, cell morphology and cell fate, regulating tissue homeostasis. Cell–cell adhesion [22,23], with cell-ECM adhesion [24] and stretch-sensitive ion channels [25] are all part of the mechanosensitive machinery used by cells to sense and respond to forces.

Adherens junctions containing E-cadherin are also key players in mechanotransduction mechanisms, contributing to balance the force distribution between sites of cell–cell attachment [26]. Since adherens junctions are linked to actin cytoskeleton, they are able to sense mechanical stimuli and transduce and distribute forces generated from the intercellular attachment, eventually affecting cell behavior [27].

Regarding the molecular events underlying mechanotransduction, the pivotal role played by the proteins yes-associated protein (YAP) and transcriptional coactivator with PDZ-binding motif (TAZ), two key transducers of mechanical signals, has been reported. They are transcriptional activators that shuttle between the cytoplasm and the nucleus, interacting with other transcription factors and influencing genes that control cell proliferation [28]. YAP/TAZ are reported to be involved in EMT since they can induce changes in the phenotype of tumor cells and produce EMT depending on their gene expression levels and cellular context, creating a bidirectional link to maintain the EMT genetic program [29].

The biological effects of mechanotransduction and mechanical forces on cells’ behavior are not only important in the context of normal tissue physiology, but it is now well known that they also exert a profound impact on cancer cells, impacting proliferation, metastatization capability and resistance to therapies.

In normal pancreas, YAP expression is limited to centroacinar and ductal cells, whilst they become up regulated in pancreatic intraepithelial neoplasia (PanIN) and pancreatic cancer cells, as well as in the activated pancreatic stellate cells located in the microenvironment in both chronic pancreatitis and pancreatic cancer [30,31,32]. The YAP gene has been proposed as a candidate oncogene involved in the progression and metastatic potential of PDAC [33], and the abrogation of progression from early PanIN to PDAC was observed in a YAP knockout mouse model. YAP activity represents a key point of convergence signaling pathways, mechanical cues and cell density, all of which play a critical role in PDAC development [29], so that it could represent a possible therapeutic target in PDAC and the understanding of how YAP could impact PDAC cell biology and behavior could be pivotal to finding new strategies to treat PDAC. Indeed, lipophilic statins were recently reported to restrain YAP activity and proliferation in pancreatic cancer cell models in vitro and to attenuate early lesions leading to PDAC in vivo [34].

In previous studies the EMT-related phenotype of PDAC cells was characterized by evaluating the expression of the main EMT markers. In the present study, we attempted to unravel the bidirectional relationship between PDAC cell characteristics and the response to mechanical cues. We investigated how cell characteristics per se can influence the expression of mechanosensors and, in turn, how mechanical cues can be involved in influencing cell phenotype. This study is therefore aimed at the in vitro characterization of the intricate interplay between mechanical cues and YAP expression in PDAC cells having different EMT-related phenotypes. For this purpose, a panel of PDAC cell lines, representative of more “epithelial” or “mesenchymal” phenotype, were cultured in 2D and 3D spheroids and analyzed by morphological and molecular approaches after cytochalasin B (CyB) treatment.

## 2. Materials and Methods

### 2.1. Cell Cultures

The pancreatic cancer cell lines HPAC, BxPC-3 and MIA PaCa-2 from pancreatic ductal adenocarcinoma (PDAC) were obtained from the American Type Culture Collection (American Type Culture Collection, ATCC) (Manassas, VA, USA).

HPAC and MIA PaCa-2 cells were cultured in Dulbecco’s Modified Eagle’s Medium (DMEM) and BxPC-3 in RPMI supplemented with 10% heat-inactivated fetal bovine serum (FBS), 2 mM glutamine, antibiotics (100 U/mL penicillin, 0.1 mg/mL streptomycin), and 0.25 μg/mL amphotericin B (Euroclone, Pero, Milan, Italy). Cell viability was determined by Trypan blue staining. Cells were cultured in T25 flasks at 37 °C in a humidified atmosphere containing 5% CO_2_.

To obtain 3D-spheroids, PDAC cells (5 × 10^3^ cells) were seeded in 24-well multiwell plates coated with 1% agarose in cell culture medium. After 3 days, spheroid formation and integrity were verified by phase-contrast microscope. For morphological and molecular evaluations, spheroids were harvested after 7 days. Duplicate samples of PDAC cells grown in a 2D-monolayer and in 3D-spheroids were analyzed.

### 2.2. Cytochalasin B Treatment

To analyze the involvement of mechanical forces in cell biology, PDAC cells grown in 2D monolayers or 3D spheroids were treated with 10 µM cytochalasin B (CyB) (Santa Cruz Biotechnology, Dallas, TX, USA), an inhibitor of actin filaments polymerization. The dose of CyB used to treat PDAC cells was used in previous studies [35,36]. Cells treated with CyB or left untreated were harvested after 48 h.

### 2.3. Western Blot

Whole cell lysates were prepared in a lysis buffer containing Tris-HCl 50 mM pH 7.6, 150 mM NaCl, 1% Triton X-100, 5 mM EDTA, 1% Sodium Dodecyl Sulphate (SDS), proteases inhibitors, and 1 mM sodium orthovanadate to inhibit phosphatases. After a 30 min incubation on ice, lysates were centrifuged at 14,000× *g* for 10 min at 4 °C to remove cell debris. For Western Blot analysis, 25 µg of total proteins were diluted in SDS sample buffer, loaded on 10% SDS polyacrylamide gel, run under reducing and denaturing conditions at 80 V according to Laemmli, and transferred at 90 V for 90 min at 4 °C to a nitrocellulose membrane in 0.025 M Tris, 192 mM glycine, and 20% methanol, pH 8.3. To analyze E-cadherin and VNC expression, membranes were incubated for 1 h at room temperature with monoclonal antibodies to E-cadherin (1:2500, Becton Dickinson, Milan, Italy) and VNC (1:2000) (clone VIN-11-5, Biotechne, Milan, Italy) and, after washing, in horseradish peroxidase (HRP)-conjugated rabbit anti-mouse antibody (1:20,000 dilution, Merck, Milan, Italy).

To analyze YAP and p-YAP expression, membranes were incubated with the YAP (D8H1X) XP^®^ Rabbit mAb and p-YAP (S109) Rabbit Ab (Cell Signaling Technology, Danvers, MA, USA). After the incubation with a horseradish peroxidase (HRP)-conjugated goat anti-rabbit antibody (1:20,000 dilution, Cell Signaling Technology), immunoreactive bands were revealed using the Amplified Opti-4CN (Bio Rad, Segrate, Milan, Italy).

To confirm equal loading, membranes were reprobed by monoclonal antibody to α-tubulin (1:2000 dilution, Sigma Aldrich, St. Louis, MO, USA).

### 2.4. Immunofluorescence

The expression and localization of E-cadherin, vimentin, vinculin, YAP/TAZ, p-YAP and actin cytoskeleton organization were analyzed by fluorescence microscopy. For this purpose, PDAC cells were grown in 2D monolayers on 12 mm diameter rounded coverslips or in 3D spheroids.

Cells and 3D spheroids were washed in phosphate-buffered saline (PBS), fixed in 4% paraformaldehyde in PBS-containing 2% sucrose for 10 or 30 min, respectively, at room temperature, post-fixed in 70% ethanol, and stored at −20 °C until use. They were incubated with the primary monoclonal antibodies mouse anti-E-cadherin (1:2500, Becton Dickinson), mouse anti-vimentin (1:200, Novocastra, Leica Microsystems, Milan, Italy), mouse anti-vinculin (1:300, Novus, Shanghai, China), rabbit YAP/TAZ (1:400, D24E4, Cell Signaling Technology) and rabbit anti-p-YAP (1:400, S109, Cell Signaling Technology). Secondary antibodies conjugated with Alexa 488 (1:500, Molecular Probes, Invitrogen, Waltham, MA, USA) were applied for 1 h at room temperature in PBS. Negative controls were incubated, omitting the primary antibody. Actin cytoskeleton was analyzed using 50 µM rhodamine-phalloidin (Sigma-Aldrich, St. Louis, MO, USA). Finally, after incubation with 4′,6-Diamidine-2′-phenylindole dihydrochloride (DAPI) (1:100,000, Sigma Aldrich), the samples were mounted on glass slides using mowiol. Cells were observed by a WD THUNDER Imager Tissue 3D (Leica Microsystems, Buccinasco, Milan, Italy).

### 2.5. Real-Time PCR

Cells grown in 2D monolayers and 3D spheroids were harvested and total RNA was (Tri-Reagent, Sigma, Milan, Italy). One µg of total RNA was reverse-transcribed in 20 µL final volume of reaction mix (Biorad, Segrate, Milan, Italy). Gene expression Slug and Snail was analyzed by real-time RT-PCR in samples run in triplicate. Gene expression was normalized on glyceraldehyde 3-phosphate dehydrogenase (GAPDH) to normalize the differences in the amount of total RNA in each sample. The primers sequences were the following: GAPDH: forward CCCTTCATTGACCTCAACTACATG, reverse TGGGATTTCCATTGATGACAAGC; Slug: forward TGTTTGCAAGATCTGCGGC, reverse TGCAGTCAGGGCAAGAAAAA; Snail: forward CTTCCAGCAGCCCTACGAC, reverse CGGTGGGGTTGAGGATCT; CTGF: forward AGGAGTGGGTGTGTGACGA, reverse CCAGGCAGTTGGCTCTAATC; CYR61: forward CCTTGTGGACAGCCAGTGTA, reverse ACTTGGGCCGGTATTTCTTC; LATS1: forward CTCTGCACTGGCTTCAGATG, reverse TCCGCTCTAATGGCTTCAGT. Each sample was analyzed in triplicate in a Bioer LineGene 9600 thermal cycler (Bioer, Hangzhou, China). The cycle threshold (Ct) was determined and gene expression levels relative to those of GAPDH were calculated using the ΔCT method.

### 2.6. SDS-Zymography

Serum-free culture media were mixed 3:1 with sample buffer (containing 10% SDS). Samples (5 μg of total protein per sample) were run under non-reducing conditions without heat denaturation on 10% polyacrylamide gel (SDS-PAGE) co-polymerized with 1 mg/mL of type I gelatin. The gels were run at 4 °C. After SDS-PAGE, the gels were washed twice in 2.5% Triton X-100 for 30 min each, and incubated overnight in a substrate buffer at 37 °C (Tris-HCl 50 mM, CaCl_2_ 5 mM, 0.02% NaN_3_, pH 7.5). MMP gelatinolytic activity, detected after staining the gels with Coomassie brilliant blue R250 as clear bands on a blue background, was quantified by densitometric scanning (UVBand, Eppendorf, Milan, Italy).

### 2.7. Statistical Analysis

Statistical analysis was performed using GraphPad Prism v 9.3 software (GraphPad Software Inc., San Diego, CA, USA). Data were obtained from two replicate experiments for each cell line in each experimental condition cultured in duplicate and were expressed as mean ± standard deviation (SD). A comparison of the groups was calculated using one-way ANOVA. Differences associated with *p* values lower than 5% were to be considered significant.

## 3. Results

### 3.1. Characterization of PDAC Cell Morphology and Phenotype in 2D and 3D Cultures

**Figure 1:**
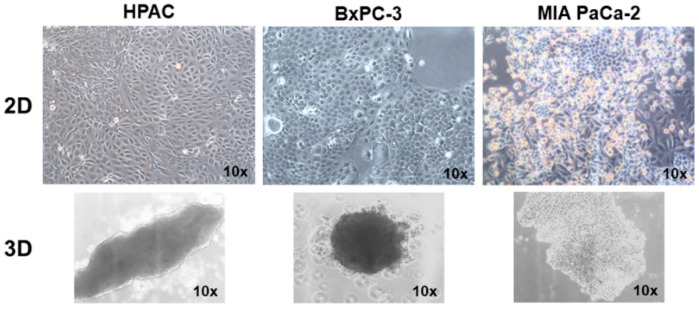
Micrographs at the phase contrast microscope showing cell morphology of PDAC cells grown in 2D monolayers and 3D spheroids. While HPAC and BxPC-3 show a more epithelial phenotype and form spheroids containing densely packed cells, MIA PaCa-2 cells on 2D monolayers reveal the presence on both epithelial and mesenchymal cells, and their spheroids are less densely packed. Original magnification 10×.

HPAC, BxPC-3 and MIA PaCa-2 PDAC cells exhibit a different phenotype. According to the ATCC description, HPAC cells are characterized by an epithelial phenotype, with cells having a polygonal shape and growing tightly apposed when cultured in 2D monolayers (Figure 1). A similar phenotype and arrangement were also observed for BxPC-3 cells. By contrast, 2D monolayers of MIA PaCa-2 cells show the co-presence of two different cell populations, some apposed epithelial-like cells, whilst some other cells were fusiform and more mesenchymal-like (Figure 1). The morphological characteristics of cells grown in 2D monolayers were maintained when cells were grown as spheroids in three-dimensional settings. Indeed, HPAC and BxPC-3 spheroids were compact and contained tightly apposed cells. By contrast, MIA PaCa-2 spheroids were bigger and flattened, with cells less tightly apposed (Figure 1).

**Figure 2:**
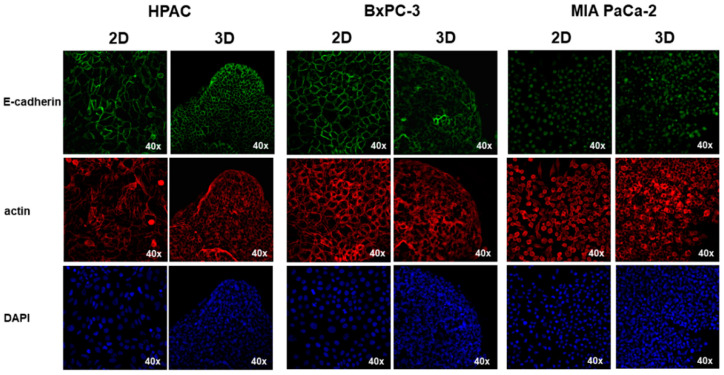
Immunofluorescence analysis for E-cadherin (green) and actin cytoskeleton (red) in PDAC cells cultured in 2D monolayers and 3D spheroids. The three PDAC cells exhibited an evident cortical actin, but E-cadherin immunoreactivity at cell membrane was detectable only in HPAC and BxPC-3 cells. Original magnification: 40×.

**Figure 3:**
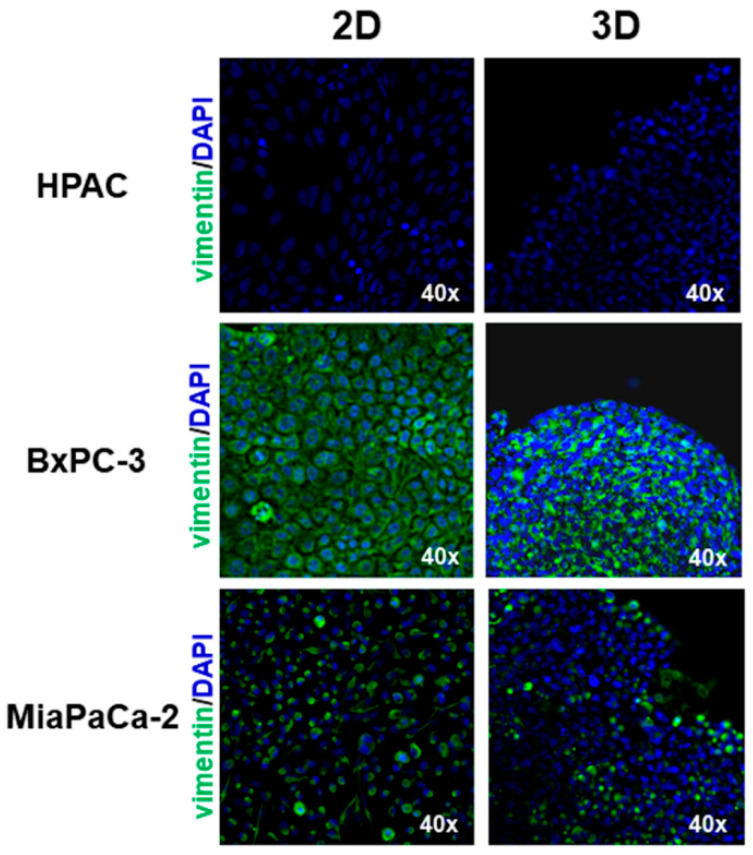
Immunofluorescence analysis for the mesenchymal marker vimentin (green) in PDAC cells cultured in 2D monolayers and 3D spheroids, showing that vimentin expression is detectable in BxPC-3 and MIA PaCa-2. Original magnification: 40×.

The epithelial phenotype of HPAC and BxPC-3 was confirmed by immunofluorescence analysis of E-cadherin and cortical actin expression. In HPAC, E-cadherin at cell boundaries and an evident cortical actin immunostaining were detected, confirming that adherens junctions and the epithelial phenotype of HPAC cells are preserved either in 2D and 3D cell cultures, as previously reported [37] (Figure 2). Accordingly, vimentin expression was undetectable (Figure 3). In BxPC-3 cells, E-cadherin and actin showed a pattern similar to HPAC cells (Figure 2) but vimentin expression was also detected (Figure 3), suggesting a hybrid phenotype. MIA PaCa-2 cells also exhibited a hybrid phenotype but were more mesenchymal compared to BxPC-3 cells. In fact, E-cadherin immunoreactivity was confined to the cytoplasm of MIA PaCa-2 cells, suggesting that adherens junctions were degraded during EMT and therefore not functional. However, cortical actin was detectable, seeming more evident in cells that had the more epithelial-like morphology (Figure 2). The mesenchymal characteristics of MIA PaCa-2 cells were also confirmed by the intense vimentin staining (Figure 3).

### 3.2. E-Cadherin and Vinculin Expression

**Figure 4:**
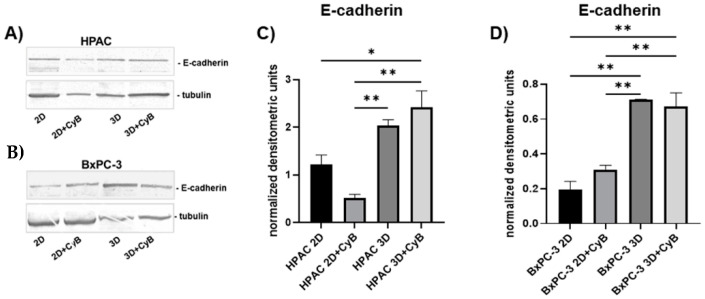
Representative Western blot showing E-cadherin expression in whole cell lysates of HPAC (**A**) and BxPC-3 cells (**B**). Bar graphs showing E-cadherin protein levels after densitometric analysis of immunoreactive bands in HPAC (**C**) and BxPC-3 cells (**D**). Data are expressed as mean ± SD. * *p* < 0.05, ** *p* < 0.01.

To deeply investigate the relationship between cell phenotype and mechanical cues in PDAC cells exhibiting different morphology and expression of EMT markers, cells cultured in 2D monolayers and 3D spheroids were treated with CyB, an inhibitor of actin cytoskeleton, to impair the role of actin cytoskeleton in mechanotransduction. Western blot analysis showed that E-cadherin was significantly up-regulated in HPAC cells grown in 3D spheroids compared to 2D monolayers (*p* < 0.05). In 2D monolayers E-cadherin tended to be expressed at a lower extent than after CyB administration, whilst it was significantly up-regulated in 3D spheroids (*p* < 0.05 for HPAC 3D + CyB vs. 2D, and *p* < 0.01 for HPAC 3D + CyB vs. 3D and 2D + CyB) (Figure 4A,C). BxPC-3 3D spheroids showed a significant up-regulation of E-cadherin compared to 2D monolayers (*p* < 0.01 for 3D vs. 2D and 2D + CyB, *p* < 0.01 for 3D + CyB vs. 2D and 2D + CyB), but CyB did not affect its expression (Figure 4B,D). The full length E-cadherin was undetectable in MIA PaCa-2 cells (data not shown), according to the immunofluorescence analysis.

**Figure 5:**
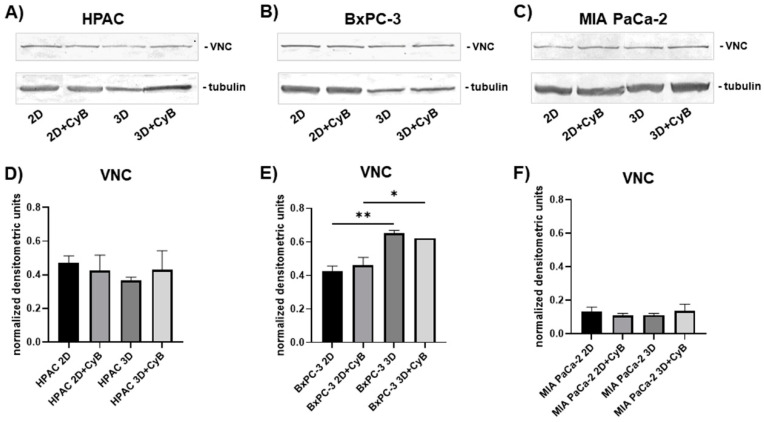
Representative Western blot showing VNC expression in whole cell lysates of HPAC (**A**), BxPC-3 (**B**) and MIA PaCa-2 cells (**C**). Bar graphs showing E-cadherin protein levels after densitometric scanning of immunoreactive bands in HPAC (**D**), BxPC-3 (**E**) and MIA PaCa-2 cells (**F**). Data are expressed as mean ± SD. * *p* < 0.05, ** *p* < 0.01.

Adherens junctions are formed by a complex of proteins connected to the actin cytoskeleton by vinculin (VNC). Western blot analysis of whole cell lysates revealed that VNC was similarly expressed in both 2D and 3D HPAC (Figure 5A,D) and MIA PaCa-2 cells (Figure 5C,F) even after CyB treatment. In BxPC-3, VNC resulted in significantly higher 3D spheroids compared to 2D monolayers in untreated (*p* < 0.01) or CyB treated cells (*p* < 0.05) (Figure 5B,E).

**Figure 6:**
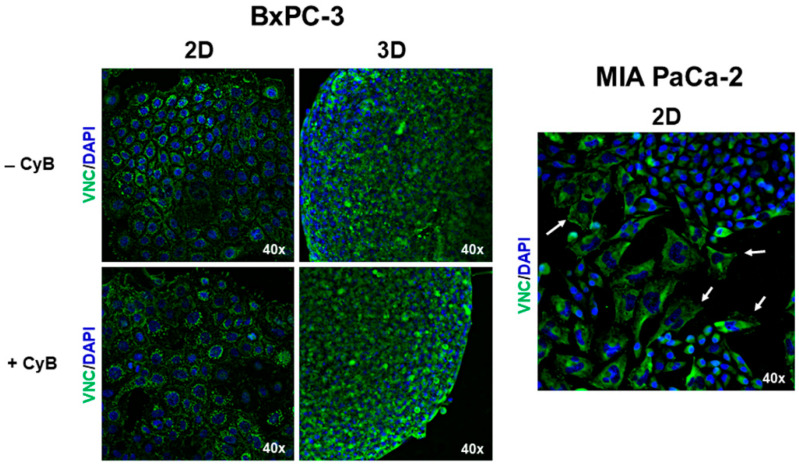
Immunofluorescence analysis for VNC (green) in BxPC-3 and MIA PaCa-2 cells. In BxPC-3 monolayers VNC is expresses at cell boundaries as well as in the cytoplasm revealing a punctate immunoreactivity. different than in 3D spheroids. In MIA PaCa-2 cells an evident VCN expression was detectable in fibroblastoid cells at the level of their lamellipodia (arrows). Original magnification: 40×.

VNC expression was analyzed by immunofluorescence in BxPC-3 and MIA PaCa-2. In BxPC-3 grown in 2D monolayers and 3D spheroids, VNC immunoreactivity has a punctate pattern at the cell boundaries where E-cadherin-containing cell junctions are located. Moreover, VNC is also evident in the cytoplasm of 2D monolayers, likely where cells are attached on the plastic substrate by focal adhesions. CyB treatment did not modify VNC expression, according to Western blot analysis (Figure 6). A different VNC organization was evident in MIA PaCa-2 cells having a more mesenchymal phenotype. In these cells, VNC was evident in lamellipodia at front of migration of the cells exhibiting a fibroblastoid morphology, while the epithelioid cell component revealed a cytoplasmic distribution (Figure 6).

Since HAPAC, BxPC-3 and MIA PaCa-2 cells exhibit a different phenotype and social behavior when grown in 3D spheroids, to better understand the effect of mechanical cues on the EMT process, the expression of the main EMT markers in untreated and CyB-treated cells was analyzed.

**Figure 7:**
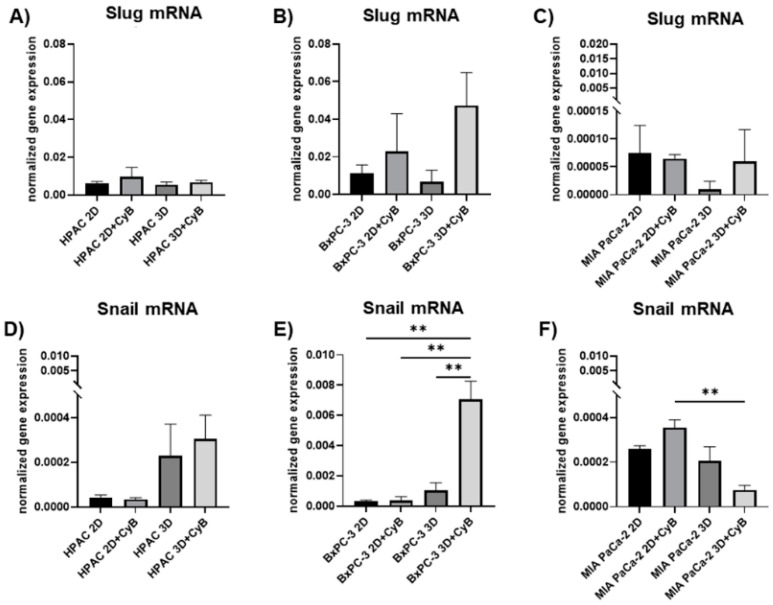
Bar graphs showing, respectively, Slug and Snail gene expression in HPAC (**A**,**D**), BxPC-3 (**B**,**E**) and MIA PaCa-2 cells (**C**,**F**) assessed by real-time PCR. Data were normalized on GAPDH gene expression and are expressed as mean ± SD for at least three independent experiments. ** *p* < 0.01.

Slug mRNA levels were not significantly affected in the different experimental conditions in all the considered cell lines (Figure 7A–C). By contrast, Snail gene expression differed in HPAC, BxPC-3 and MIA PaCa-2 cells. Although without a significant difference, Snail gene expression tended to be higher in HPAC 3D spheroids compared to 2D monolayers. However, no further increase of Snail expression was observed upon CyB (Figure 7D). BxPC-3 cells showed the most evident increase in 3D spheroids treated with CyB compared to untreated 3D spheroids (*p* < 0.01). This up-regulation was significant also comparing 2D monolayers and 3D spheroids treated with CyB (*p* < 0.01) (Figure 7E). An opposite pattern of expression was observed in MIA PaCa-2 cells. In fact, a significant down regulation of Snail was induced by CyB treatment in 3D spheroids compared to 2D monolayers (*p* < 0.01) (Figure 7F).

### 3.3. YAP and TAZ Expression

**Figure 8:**
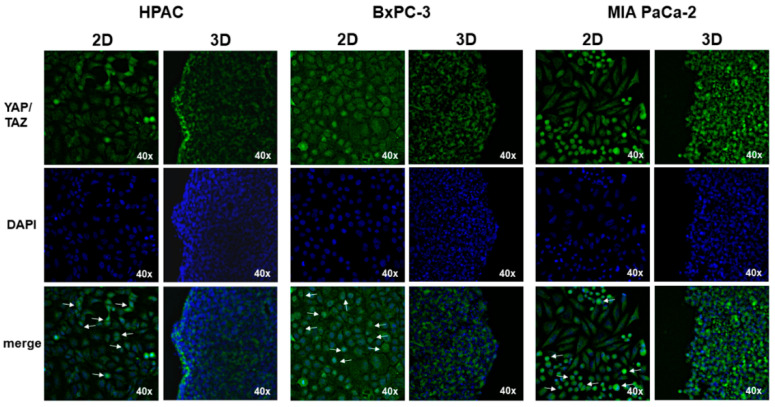
Immunofluorescence analysis for YAP/TAZ (green) in PDAC cells cultured in 2D monolayers and 3D spheroids. The merged micrographs show that HPAC and BxPC-3 cells grown in 2D monolayers exhibited a more nuclear immunolabeling compared to 3D spheroids (see arrows), having a predominantly YAP/TAZ cytoplasmic localization. Conversely, in MIA PaCa-2 cells YAP/TAZ immunoreactivity was mostly cytoplasmic in both 2D and 3D cell cultures. Original magnification: 40×.

To characterize the expression of the YAP/TAZ mechanosensors in 2D and 3D cell cultures of PDAC cells, immunofluorescence analysis was performed. HPAC and BxPC-3 cells exhibited a similar pattern of YAP/TAZ cellular distribution, showing a mostly cytoplasmic YAP/TAZ expression, although in few cells cultured in 2D monolayer a nuclear localization was also detected (Figure 8). Conversely, MIA PaCa-2 revealed a different pattern of expression, showing that in 2D monolayers the subpopulation of cells exhibiting an epithelial morphology had a more intense cytoplasmic labeling compared to the mesenchymal-like subpopulation. Immunoreactivity was mostly cytoplasmic in both 2D and 3D cell cultures (Figure 8).

**Figure 9:**
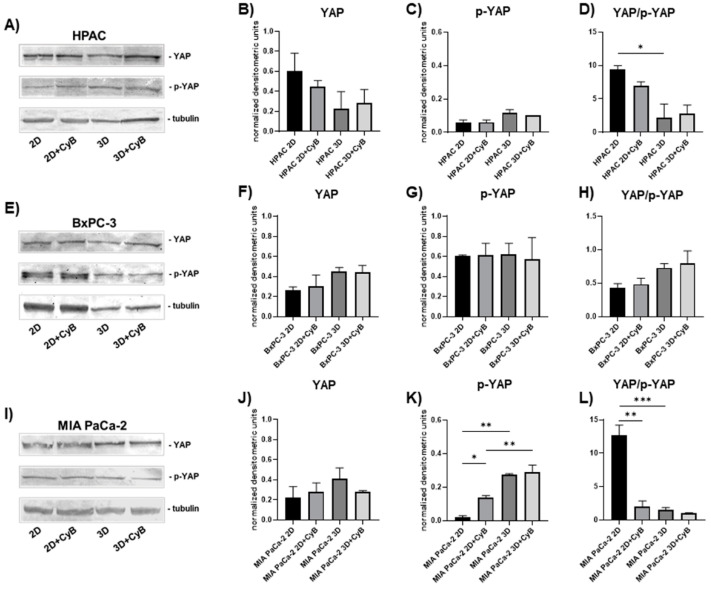
Representative Western blot showing YAP and p-YAP expression in whole cell lysates of HPAC (**A**), BxPC-3 (**E**) and MIA PaCa-2 cells (**I**). Bar graphs showing YAP, p-YAP and the YAP/p-YAP ratio, respectively, in HPAC (**B**–**D**), BxPC-3 (**F**–**H**) and MIA PaCa-2 cells (**J**–**L**). Data are expressed as mean ± SD. * *p* < 0.05, ** *p* < 0.01, *** *p* < 0.001.

To better understand whether mechanical cues could be related to cell morphology and phenotype, YAP and p-YAP expression, the active and the inactive form of YAP protein respectively, was analyzed in PDAC cells treated or not with CyB. Western blot analysis revealed a different pattern of expression in the three considered cell lines. Although in HPAC cells YAP and p-YAP levels were not significantly affected by the different experimental settings, the YAP/p-YAP ratio was significantly decreased in 3D spheroids compared to 2D monolayers (*p* < 0.05). The treatment with CyB did not exert any effect (Figure 9A–D). In BxPC-3, YAP and p-YAP remained unchanged, as well as their ratio (Figure 9E–H). In MIA PaCa-2 cells, YAP was not influenced by the experimental setting (Figure 9I,J). By contrast, we observed a significant up-regulation of p-YAP in 3D spheroids compared to 2D monolayers (*p* < 0.01) (Figure 9I,K). p-YAP was significantly up-regulated after CyB treatment in 2D monolayers (*p* < 0.05) and remained expressed to a higher extent in 3D spheroids compared to 2D monolayers after CyB administration (*p* < 0.01) (Figure 9I,K). The YAP/p-YAP ratio was significantly higher in 2D monolayers compared to 3D spheroids (*p* < 0.001) and after CyB administration (*p* < 0.01) (Figure 9I,L).

**Figure 10:**
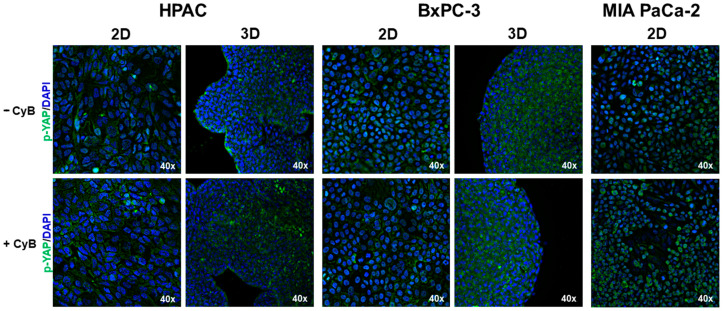
Immunofluorescence analysis for p-YAP (green) in PDAC cells cultured in 2D monolayers and 3D spheroids. The micrographs show that in HPAC and BxPC-3 cells p-YAP expression is mostly cytoplasmic in both 2D and 3D cell cultures. By contrast, MIA PaCa-2 cells grown in 2D monolayer revealed a different pattern of expression. Indeed, the more fibroblastoid subpopulation is characterized by a lower p-YAP expression, whereas the more epithelial cells revealed a more intense and cytoplasmic expression, according to the different cell adhesion on the plastic substrate. Original magnification: 40×.

To better understand how mechanical cues influence the mechanotransduction related mechanism, p-YAP was also investigated at the morphological level. Immunofluorescence analysis revealed that in HPAC and BxPC-3 cells grown in 2D monolayers the immunolabeling is mostly cytoplasmic and is not affected by CyB treatment (Figure 10). Conversely, MIA PaCa-2 cells revealed a different pattern of expression in the fibroblastoid and epithelial cell subpopulations: more fibroblastoid cells grown in 2D monolayer are attached on the plastic substrate by focal adhesions triggering mechanical inputs and, accordingly, are characterized by a lower p-YAP expression. By contrast, epithelial-like cells have a rounded-polygonal morphology and a lower adhesion on the plastic substrate that, altogether, trigger a lower mechanical stimulation consistent with a higher p-YAP expression and, therefore, a more intense cytoplasmic expression that further becomes more evident and intense after CyB administration (Figure 10), according to the Western blot analysis.

**Figure 11:**
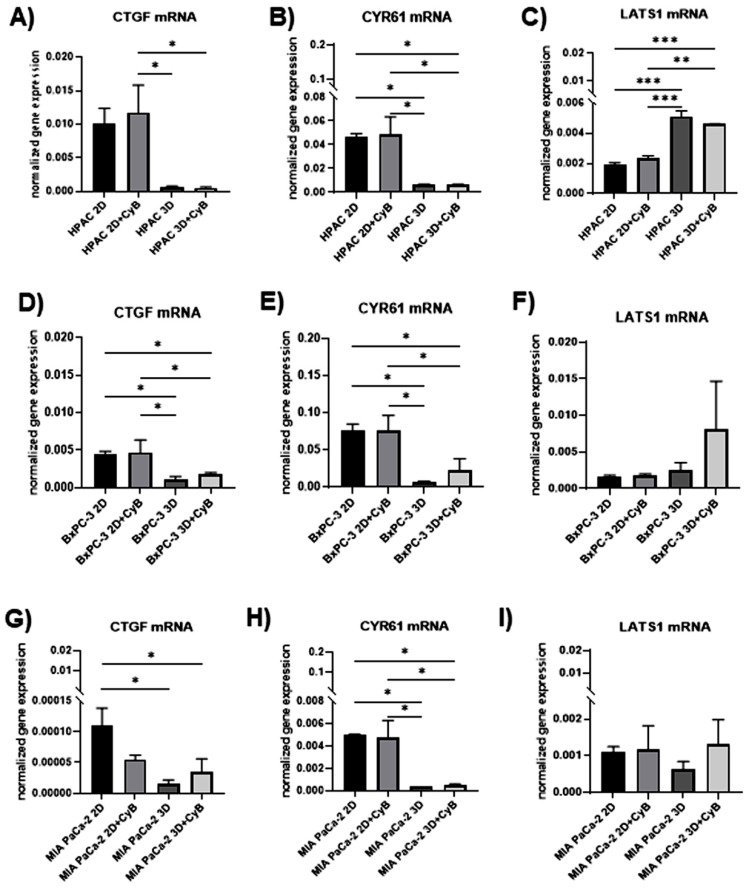
Bar graphs showing, respectively, CTGF, CYR61 and LATS1 gene expression in HPAC (**A**–**C**), BxPC-3 (**D**–**F**) and MIA PaCa-2 cells (**G**–**I**) assessed by real-time PCR. Data were normalized on GAPDH gene expression and are expressed as mean ± SD for at least three independent experiments. * *p* < 0.05, ** *p* < 0.01, *** *p* < 0.001.

To investigate how YAP expression and activity are related to PDAC cell phenotype, we analyzed the gene expression of CTGF and CYR61, which are target genes of YAP, and of LATS1, a Hippo kinase involved in YAP regulation [38]. CTGF and CYR61 gene expression resulted in being significantly down regulated in 3D spheroids compared to 2D monolayers in the three considered cell lines. In detail, a significant CTGF down regulation was detected in HPAC 3D and HPAC 3D + CyB compared to HPAC 2D + CyB (*p* < 0.05). CYR61 decreased in 3D and 3D + CyB compared to 2D + CyB (*p* < 0.05), and in 3D and 3D + CyB compared to 2D (*p* < 0.05), similarly to MIA PaCa-2 cells (Figure 11A,B).

In BxPC-3, CTGF and CYR61 significantly decreased in 3D compared to 2D and 2D + CyB (*p* < 0.05). They also resulted in being downregulated in 3D + CyB vs. 3D and 2D (*p* < 0.05) (Figure 11D,E). In MIA PaCa-2 a significant CTGF downregulation was observed in 3D and 3D + CyB compared to 2D (*p* < 0.05). CYR61 was decreased in 3D and 3D + CyB compared to 2D + CyB (*p* < 0.05), and in 3D and 3D + CyB compared to 2D (*p* < 0.05), similarly to HPAC cells (Figure 11G,H). LATS1 gene expression resulted significantly affected only in HPAC cells, showing an up-regulation in 3D and 3D + CyB compared to 2D (*p* < 0.001), and in 3D and 3D + CyB compared to 2D + CyB (*p* < 0.01 and *p* < 0.001, respectively).

### 3.4. Invasive Potential

**Figure 12:**
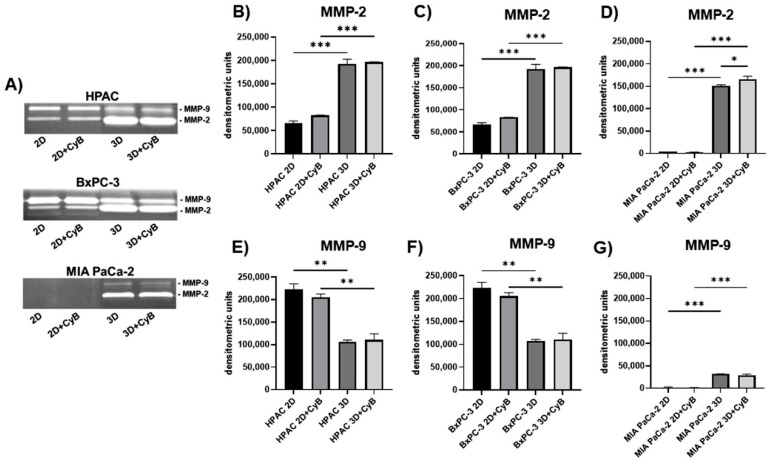
(**A**) Representative SDS-zymography showing MMP-2 and MMP-9 activity assayed in serum-free cell supernatants of PDAC cells. Bar graphs showing MMP-2 and MMP-9 activity after densitometric analysis of lytic bands, respectively, in HPAC (**B**,**E**), BxPC-3 (**C**,**F**) and MIA PaCa-2 cells (**D**,**G**). Data are expressed as means ± SD. * *p* < 0.05, ** *p* < 0.01, *** *p* < 0.001.

The invasive potential of PDAC cells was evaluated by analyzing MMP-2 and -9 activity in cell supernatants by SDS-zymography. Zymograms revealed a similar pattern of MMP-2 expression in the considered PDAC cells. Indeed, MMP-2 activity was significantly higher in 3D spheroids compared to 2D monolayers in HPAC, BxPC-3 and MIA PaCa-2 supernatants (*p* < 0.001). This up-regulation was also maintained upon CyB treatment (*p* < 0.001) (Figure 12A–C). CyB treatment further increased MMP-2 levels in MIA PaCa-2 3D spheroids (*p* < 0.05) (Figure 12D).

MMP-9 activity displayed an opposite pattern of expression in cells having a more epithelial than mesenchymal phenotype. Indeed, it resulted in being significantly down-regulated in HPAC and BxPC-3 3D spheroids compared to 2D monolayers (*p* < 0.002), and the down-regulation was maintained after CyB treatment (*p* < 0.01) (Figure 12A,E,F). In MIA PaCa-2 supernatants MMP-9 activity had an inverse expression compared to epithelial-like cells. Accordingly, it was almost undetectable, but resulted in being up-regulated in 3D spheroids compared to 2D monolayers (*p* < 0.001). This up-regulation was maintained after CyB administration (*p* < 0.001) (Figure 12A,G).

Overall, these findings suggest that MMPs expression is mostly influenced by the 3D arrangement. Moreover, in MIA PaCa-2 cells, characterized by more mesenchymal phenotype, the actin cytoskeleton appears to be involved in the modulation of the invasive potential mediated by MMP-2.

**Figure 13:**
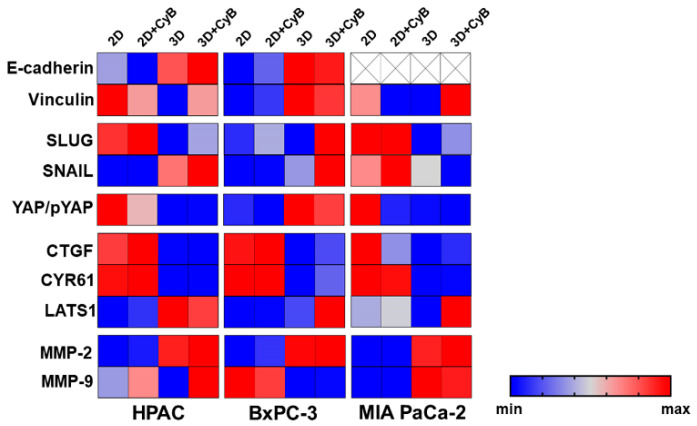
Heatmap summarizing all the results relative to the analysis of PDAC cells in the different experimental conditions. The bar on the right shows how colors map to numeric values.

The overall results are summarized in Figure 13 allowing for a comparison of the different patterns characterizing HPAC, BxPC-3 and MIA PaCa-2 cells.

## 4. Discussion

In the present study, we attempted to investigate the complex interplay between mechanotransduction and the acquisition of a specific phenotype by PDAC cells. Although many aspects of the biological effects of mechanical cues on cancer cells are still obscure and need to be fully elucidated, there are many insights indicating that mechanical forces have a profound impact on tumors, including pancreatic cancer, able to affect proliferation, metastatization and response to therapies.

We utilized three different PDAC cell lines representative of different possible phenotypes found in pancreatic cancers. HPAC and BxPC-3 grown in 2D monolayers are characterized by the typical features of epithelial cells showing the well detectable immunoreactivity of functional E-cadherin-containing adherens junctions at cell boundaries and cortical actin. However, the expression of vimentin by BxPC-3 cells, although they possess more “epithelial” characteristics, indicates that these cells exhibit a hybrid phenotype. By contrast, MIA PaCa-2 cells grow in 2D monolayers revealing two distinct cell populations: small rounded-polygonal epithelial cells growing apposed and exhibiting an evident cortical actin, and spindle-like elongated fibroblastoid cells growing without evident cell junctions. In MIA PaCa-2 cells, E-cadherin was almost undetectable and eventually showed a cytoplasmic localization consistent with not functional adherens junctions. The phenotype we observed is perfectly in line with that reported on the ATCC website (Available online: http://www.atcc.org, accessed on 15 February 2022). These characteristics influenced the formation and the morphology of 3D spheroids. Indeed, HPAC and BxPC-3 3D spheroids were compact and contained densely packed and apposed cells, whilst cells in MIA PaCa-2 3D spheroids were less densely apposed and exhibited a low cell–cell adhesion. Phenotypic differences were also evident in relation to the invasive potential. In fact, while all the three cell lines showed similar MMP-2 activity, MIA PaCa-2 had an opposite pattern of expression of MMP-9 in 2D and 3D cell cultures, compared to the epithelial HPAC and BxPC-3 cells. We can hypothesize that the differences in MMP-2 and MMP-9 expression could also be related to the intrinsic phenotypic characteristics of PDAC cells. In fact, HPAC and BxPC-3, more “epithelial”, can invade their surrounding microenvironment through collective migration [39], exploiting the release of MMPs from invadopodia [40]. Conversely, MIA PaCa-2 cells, more “mesenchymal”, possibly invade in a single cell mechanism [39].

E-cadherin is a pivotal determinant of the epithelial phenotype. Contacts at cell–cell junctions are not only needed to maintain tissue integrity, but also act as cellular devices able to integrate subcellular forces across neighboring cells and respond to tension, thereby influencing cell behavior. When cells are cultured in 2D monolayers and 3D spheroids they experience a different “environment” and “sociality”. The extent of cell–cell and cell–matrix adhesions greatly differ in the two culture settings and, consequently, the mechanical forces applied on cancer cells. This aspect has a profound rebound on cell behavior and biological properties.

Cells cultured in 2D monolayers grow on a large stiff surface and, therefore, they have the possibility to develop focal adhesions and evident stress fibers. Conversely, in 3D cultures, cells are engaged in cell–cell junctions on their entire surface, so that the intercellular forces are higher compared to 2D monolayers. Moreover, 3D spheroids contain smaller and more rounded cells, a morphology that allow to increase cell–cell contacts with neighboring cells that involve the entire cell surface, engaging a different number of cell–cell junctions extending on the entire cell surface. Moreover, cell geometry should also be considered. In fact, it was demonstrated that cells grown on a small surface on small islands experience a lower mechanical stimulation compared to cells [38,41]. Finally, 3D spheroids experience a less stiff environment since they are floating in the cell culture medium and do not exhibit focal adhesions. Despite that, when cells are grown in 3D spheroids, the intercellular forces are higher compared to the forces acting at cell–matrix or cell–substrate adhesions as in 2D monolayers. Therefore, cells cultured in these two experimental conditions can experience different forces and, consequently, different mechanical stimulation.

In this study, we were interested in understanding how PDAC cell morphology and a well-defined EMT-related phenotype per se could differently impact the expression of mechanosensors and cell behavior, but also how mechanical cues could be involved in determining PDAC cell phenotype. Adherens cell junctions, in fact, can trigger intracellular forces since they pull the apposed cells. Moreover, cortical actin in “epithelial” cells or stress fibers in more “mesenchymal” cells represent two different cytoskeleton arrangements that are responsible for the different ability to respond to mechanical cues. For this purpose, 2D and 3D cultures were treated by cytochalasin B (CyB), an inhibitor of actin cytoskeleton [42], to inhibit the role of actin cytoskeleton in cell mechanotransduction.

First, we analyzed E-cadherin expression in whole cell lysates and the full length E-cadherin resulted in being significantly up-regulated in HPAC and BxPC-3 3D spheroids, compared to 2D monolayers, according to the notion that a higher cell–cell adhesion is needed for collective migration [39], as previously reported [43]. Moreover, adherens junctions contain E-cadherin trigger pulling forces acting on the apposed cells. The full length E-cadherin was undetectable in MIA PaCa-2, according to their more mesenchymal phenotype. CyB administration did not influence the E-cadherin pattern of expression, pointing to a major role played by cell arrangement and 3D architecture in influencing cell behavior. Cell–cell contacts at cell junctions are not only pivotal to maintaining tissue integrity, but should also be considered for their ability to integrate subcellular forces across neighboring cells and respond to tension, thereby influencing cell behavior. Indeed, adherens junctions are formed by a complex of proteins, including α-catenin, connected to the actin cytoskeleton by VNC. α-catenin not only stabilizes F-actin binding but, upon tensional stimulation, also recruits VNC and additional actin-binding proteins to the cell–cell contacts, such as α-actinin, formin 1, and afadin [22]. VNC stabilizes α-catenin and contributes to prolonging the tension-dependent reinforcement of the adherens junction [44]. We observed higher VNC in HPAC and BxPC-3 cells compared to MIA PaCa-2 cells. In HPAC cells, its expression remained unchanged in 2D and 3D cultures. However, it was significantly up-regulated in BxPC-3 3D spheroids compared to 2D monolayers, suggesting a recruitment of VNC to stabilize and reinforce adherens junctions induced by 3D arrangement. These data lead to the hypothesis that BxPC-3 cells grown in 3D spheroids experience higher intercellular forces at cell–cell adherens junctions that need higher levels of VNC to be stabilized. The lower VNC expression observed in MIA PaCa-2 cells is perfectly consistent with the undetectable functional adherens junctions.

The EMT marker Snail showed a different expression in PDAC cells. While Slug remained unaffected in the different experimental conditions, Snail showed an opposite expression in cells exhibiting a more epithelial phenotype. Indeed, HPAC and BxPC-3 cells showed higher Snail mRNA levels when grown in 3D spheroids, compared to 3D MIA PaCa-2 cells, characterized by fibroblastoid-mesenchymal characteristics and lower Slug gene expression. We also observed that the expression of the considered genes is not affected by CyB treatment, a finding in line with the hypothesis that actin cytoskeleton dynamics are not key players in influencing these EMT markers.

We also evaluated the expression of YAP/TAZ, since these proteins are key mechanotransducers of cell structural characteristics such as polarity, shape and activation.

Moreover, YAP is involved in the initiation and progression of several carcinomas [45,46,47,48,49,50,51,52], including pancreatic cancer [33]. It has been proposed as a candidate oncogene, and its signaling pathway can regulate EMT and cancer stemness, promoting metastasis. Furthermore, YAP was indicated as a regulator of pancreatic cancer cell motility and invasion [53].

By immunofluorescence analysis, we found that in HPAC and BxPC-3 cells cultured in 2D monolayers, YAP/TAZ was more frequently localized at a nuclear level compared to 3D spheroids. Interestingly, a similar distribution pattern was observed in the MIA PaCa-2 cell line but was only restricted to cells exhibiting an epithelial morphology and not in those having the mesenchymal morphology (Figure 7). When PDAC cells were grown in 3D spheroids, YAP/TAZ expression was predominantly cytoplasmic, consistent with the lower mechanical stimulation experienced by cells cultured in the two different experimental conditions. Indeed, the interaction of cells with a stiff plastic substrate is able to trigger YAP/TAZ activation and nuclear localization [38]. They experience intercellular forces developed by cell–cell junctions as well as cell–substrate forces developed at focal adhesions via integrins. By contrast, 3D spheroids are floating in the cell culture medium and are characterized by a lower mechanical stimulation, that can be comparable to soft substrate, leading to YAP/TAZ inactivation and cytoplasmic relocalization [38].

We then treated PDAC cells with CyB to analyze how mechanical forces impact cellular mechanoresponse. For this purpose, we evaluated YAP and p-YAP protein levels. Our data indicate that, in PDAC cells exhibiting an epithelial phenotype, YAP and p-YAP were not affected by 3D arrangement nor by CyB treatment. However, the YAP/p-YAP ratio was significantly lower in HPAC 3D spheroids compared to 2D monolayers, while it remained unchanged in BxPC-3 cells. Surprisingly, in MIA PaCa-2 cells both 3D arrangement and CyB treatment led to YAP inactivation. However, the YAP/p-YAP ratio was similar to that observed in HPAC, showing a decrease in 3D-spheroids, and after CyB treatment of 2D monolayers. These results seem consistent with a lower YAP activity in 3D spheroids and confirm that cells grown in 3D spheroids experience lower mechanical inputs leading to YAP phosphorylation. This hypothesis was confirmed by p-YAP immunofluorescence analysis, revealing a mostly cytoplasmic immunoreactivity, which was more evident when cells were grown into 3D spheroids. This effect was more evident in MIA PaCa-2 cells that are more mesenchymal, also after inhibiting actin cytoskeleton by CyB. Interestingly, an effect of CyB treatment on MMP-2 levels was observed only in these cells.

Consistently, the gene expression of CTGF and CYR61, markers of YAP transcriptional activation [38], resulted in being significantly down-regulated in PDAC 3D spheroids compared to 2D monolayers, indicating a lower mechanical stimulation. However, the inhibition of actin cytoskeleton did not seem to significantly affect their expression in both experimental conditions. This hypothesis is consistent with the observation that the phosphorylation of YAP did not increase in MEC and MSC treated with cytoskeleton inhibitors [41].

This result suggests that, in PDAC cells, the inactivation of YAP by phosphorylation is a complex mechanism involving different levels of regulation besides mechanical cues, and that the role of actin cytoskeleton has a negligible involvement in this process. Accordingly, YAP is a major downstream effector in the Hippo signaling pathway that is enrolled in different cellular mechanisms related to tissue repair, cell regeneration, proliferation and apoptosis [54]. The Hippo pathway includes a kinase cascade resulting in the inactivation by phosphorylation of YAP and in its cytoplasmic localization, thus preventing YAP translocation to the nucleus and its activity as a transcriptional co-activator. By contrast, the dephosphorylated YAP can translocate to nucleus and bind to transcriptional enhanced associate domain (TEAD) proteins leading to TEAD-mediated transcription of the downstream genes and thus regulating the expression of the target genes [54,55,56]. The inactivation of YAP by phosphorylation is mediated by kinases of the Hippo cascade, such as Mst1/2 and LATS1/2. Increased LATS1 is consistent with the inactivation of YAP transcriptional activity. LATS1 gene expression show a very different pattern in the considered PDAC cells. In fact, LATS1 resulted in being significantly up-regulated by 3D arrangement in HPAC cells, while it was not significantly affected in BxPC-3 and MIA PaCa-2 cells. Interestingly, although LATS1 is differently involved in PDAC cells having a different EMT-related phenotype, in these cells, CTGF and CYR61 exhibited a similar pattern. Moreover, LATS1 gene expression was not influenced by CyB treatment, suggesting again the marginal role of actin cytoskeleton in the observed biological process.

Although CyB is widely used in in vitro studies to analyze the involvement of actin cytoskeleton [35,36], it should be considered that CyB can impact different cellular processes, such as the induction of extracellular vesicles promoting angiogenesis [57] and the inhibition of autophagosome formation in autophagy [58], a process also involved in EMT [59]. This might represent a limitation of the present study, and further investigation is needed to finely dissect the overall effect of CyB on PDAC cell biology.

In this extremely complex scenario, our findings can be explained in the light of previous results reporting that the regulation of YAP by mechanical cues and the Hippo pathway act as two parallel inputs that act in parallel but are not exclusive and, instead, can converge and intersect [38]. In this interaction, however, the effect on YAP regulation is dependent on a mechanically competent cytoskeleton [41].

## 5. Conclusions

Overall, we can hypothesize that the different pattern observed in this study could be related both to cell arrangements as well as to the different intrinsic characteristics of the studied PDAC cells. In fact, cells experience pulling forces coming from neighboring cell or from the extracellular environment such as ECM or the substrate where they grow.

When cells grow in 2D monolayers, they use a large surface to attach to the substrate, receiving mechanical inputs from focal adhesions and adherens cell junctions that can cooperate and, at the same time, can have an antagonistic relationship. In fact, they are both connected to the actin cytoskeleton and involved in mechanotransduction mechanisms, playing a key role in balancing the force distribution between sites of cell–cell and cell–substrate attachment [26]. It was suggested that adherens junctions and focal adhesions can cross-talk since they share some mechanosensitive components [26,60]. In PDAC cells grown in 2D monolayers, YAP is mostly nuclear and its transcriptional activity is higher compared to 3D spheroids, consistently with a more intense mechanical stimulation. However, CyB treatment of 2D monolayers did not exert any evident effect leading to the hypothesis that, although CyB stabilized actin filaments, cells experienced intense mechanical forces. Since MIA PaCa-2 cells do not possess functional E-cadherin containing cell junctions and exhibit a mesenchymal-like morphology, these mechanical forces are likely derived from focal adhesions more than from cell junctions.

When cells grow in 3D spheroids they have a completely different “social” behavior since they experience an “all around” cell–cell contact, increasing intercellular cell adhesion. In these conditions cell–cell contact at cell junctions was demonstrated to activate the Hippo pathway [41,61] leading to YAP inactivation. Moreover, their crowding reduces cell size, leading to YAP inactivation [41]. In this condition, the effect of the intersection of mechanical cues and Hippo pathways could differently impact cell behavior, such as the invasive potential, depending on cell characteristics.

HPAC has an epithelial phenotype with higher E-cadherin expression when grown in 3D spheroids. The significantly decreased YAP/p-YAP ratio in 3D HPAC is paralleled by a decreased YAP transcriptional activity but CyB did not affect them. Since LATS1 exhibited and inverse pattern, we can speculate that the intracellular forces based on the increased cell–cell adhesion in 3D HPAC could activate the Hippo pathway that prevails on mechanical cues in inhibiting YAP.

In MIA PaCa-2, having a more mesenchymal phenotype and less cell–cell adhesion, the YAP/p-YAP ratio was significantly reduced by actin cytoskeleton inhibition and 3D arrangement. This can be explained considering that these fibroblastoid cells grown in 3D spheroids lose focal adhesions on the substrate and experience lower mechanical inputs. In MIA PaCa-2 cells, therefore, mechanical cues likely prevail on the Hippo pathways in YAP regulation, and this suggestion is consistent with the significant increase of MMP-2 activity in 3D spheroids after CyB treatment.

In BxPC-3, the YAP/p-YAP ratio remained unchanged while YAP transcriptional activity was significantly reduced by 3D arrangement, but not after CyB treatment. Unexpectedly, even if these cells have evident functional adherens junctions, LATS1 is not significantly affected and, possibly, its stimulation by Hippo does not prevail on actin cytoskeleton-mediated mechanical cues. The LATS1 effect, however, depends on a mechanically competent cytoskeleton [38], and its expression can be very variable in different cancer cells.

Thus, the understanding of the role of YAP in the modulation of PDAC in response to mechanical cues and different signaling pathways converging on cells to a different extent and possibly at different times needs further investigation in order to find new potential therapies for PDAC.

## Data Availability

Not applicable.
